# Nitrogen, Phosphorus and Sulfur Co-Doped Pyrolyzed Bacterial Cellulose Nanofibers for Supercapacitors

**DOI:** 10.3390/nano10101912

**Published:** 2020-09-25

**Authors:** Zheng Li, Yaogang Wang, Wen Xia, Jixian Gong, Shiru Jia, Jianfei Zhang

**Affiliations:** 1 Key Laboratory of Advanced Textile Composites of Ministry of Education, School of Textiles Science and Engineering, Tiangong University, Tianjin 300387, China; e158263980@163.com (Y.W.); or gongjixian@tiangong.edu.cn (J.G.); or zhangjianfei@tiangong.edu.cn (J.Z.); 2 Beijing Tongyizhong New Material Technology Corporation, No.17, Jingsheng South 2nd Street, Majuqiao Town, Tongzhou District, Beijing 101102, China; Xwtjpu@126.com; 3 Key Laboratory of Industrial Fermentation Microbiology, Ministry of Education, Tianjin University of Science and Technology, Tianjin 300457, China; jiashiru@tust.edu.cn

**Keywords:** pyrolyzed bacterial cellulose, nitrogen/phosphorus/sulfur co-doped nanofibres, specific capacitance, supercapacitor

## Abstract

Heteroatom doping is an effective way to raise the electrochemical properties of carbon materials. In this paper, a novel electrode material including nitrogen, phosphorus, and sulfur co-doped pyrolyzed bacterial cellulose (N/P/S-PBC) nanofibers was produced. The morphologies, structure characteristics and electrochemical performances of the materials were investigated by Scanning electron microscopy, Fourier transform infrared spectra, X-ray diffraction patterns, X-ray photoelectronic spectroscopy, N_2_ sorption analysis and electrochemical measurements. When 3.9 atom% of nitrogen, 1.22 atom% of phosphorus and 0.6 atom% of sulfur co-doped into PBC, the specific capacitance of N/P/S-PBC at 1.0 A/g was 255 F/g and the N/P/S-PBC supercapacitors’ energy density at 1 A/g was 8.48 Wh/kg with a power density of 489.45 W/kg, which were better than those of the N/P-PBC and N/S-PBC supercapacitors. This material may be a very good candidate as the promising electrode materials for high-performance supercapacitors.

## 1. Introduction

The remarkable progress in portable electronics achieved in recent decades has undoubtedly been driven by rechargeable energy storage systems such as supercapacitors, lithium-ion batteries, and others [1]. Supercapacitors is an essential reversible storage and releaser of electricity technology [2]; it can be divided into an electrochemical double-layer capacitor and pesudocapacitor, while the pesudocapacitor usually reveals a higher capacitance because of the Faradaic process from redox reaction [3]. Carbon-based supercapacitors have been considered as preferable alternative devices to replace traditional energy storage systems on account of their multiple advantages, including low weight and cost, high power delivery, and long cycling stability [4,5,6]. Heteroatom-doped carbon materials also have been an efficient method to enhance the pseudocapacitance effects, and the fabrication process is relatively easy to handle, versatile, and general [7].

Carbon nanomaterials, mainly including carbon nanoparticles [8,9], carbon nanofibers [10], grapheme [11,12] and other nanoporous carbons [13,14], have played an important role in electrode materials. It is worth noting that the three-dimensional structure is considered to have greater application potential. Bacterial cellulose (BC) is fabricated by some bacteria, and attracts considerable attention due to its low cost, sufficient porosity, and high mechanical strength, as well as an extremely fine and pure fibre network structure [15]. Pyrolyzed bacterial cellulose (PBC) possess extraordinary mechanical stability, corrosion resistance, tunable surface functionalities and transport property [16], therefore they can be used as promising electrode materials in electric double-layer capacitors (EDLCs) [17], lithium ion battery anode materials [18], catalyst carriers [19] and electric devices [20].

At present, the most commonly used heteroatoms include N, P, and S [21,22]. The introduction of nitrogen (N) atoms into sp^2^-hybridized carbon (C) frameworks is effective in modifying their electrical properties and chemical activities, which are achieved by influencing the spin density and charge distribution of the neighbor C atoms [23]. Sulfur is easy to polarize due to large lone pairs that could change the spin density of the neighboring C atoms and generate structural or chemical defects [23]. Carbon materials doped with phosphorus or sulfur elements do not show a comparable activity as nitrogen doping [24]. However, the co-doping of phosphorus/nitrogen, sulfur/nitrogen also showed an improved activity due to the enhanced asymmetry of the spin density or electron transfer on the basal plane of carbon materials, and decrease the energy gap between the highest occupied molecular orbital and the lowest unoccupied molecular orbital of the carbon materials [25,26].

Herein, heteroatom-doped pyrolyzed bacterial cellulose carbon nanofibers were fabricated via a one-step pyrolysis synthesis approach. Specifically, nitrogen, phosphorus, sulfur co-doped pyrolyzed bacterial cellulose (N/P/S-PBC) was successfully prepared by impregnating NH_4_H_2_PO_4_ and (NH_4_)_2_SO_4_ into the BC pellicle, followed by carbonization in an inert atmosphere at 800 °C. The impact of heteroatom doping on the electrochemical activities was systematically studied. Here, the results indicate that the N/P/S-PBC electrode material of supercapacitor devices will show a high energy density and excellent cycling stability.

## 2. Materials and Methods

### 2.1. Chemicals

Bacterial cellulose was purchased from Hainan Yide Food Industry co., Ltd., Haikou, China. All other reagents were commercially available from Tianjin Chemical Reagent co., Ltd. (Tianjin, China), and used as received without further purification.

### 2.2. Preparation of Heteroatom-Doped PBC

The purified BC pellicles was firstly neutralized with deionized water and then sliced into roundness (*d* = 20 mm) with a puncher. After this, the slices of BC were soaked in 100 mL NH_4_H_2_PO_4_, (NH_4_)_2_SO_4_ and NH_4_H_2_PO_4_/(NH_4_)_2_SO_4_ aqueous solution with different concentration, respectively. After that, the BC slices were oscillated at room temperature for 10 h and frozen in refrigerator. Subsequently, the slices were freeze-dried in a freeze dryer to evaporate the solvent. Finally, the as-obtained BC slices were heated in a nitrogen atmosphere at 2.0 °C/min to 520 °C and kept at this temperature for 1.0 h and then at 5.0 °C /min to 800 °C, and kept at that temperature for 1.0 h to form the pyrolyzed bacterial cellulose products (PBCs).

The doping content of heteroatom in the PBCs is controlled by adjusting the molarity of the NH_4_H_2_PO_4_ or (NH_4_)_2_SO_4_ aqueous solution. The designation of samples was listed in Table 1.

### 2.3. Characterization of Materials

SEM images were performed on a Hitachi S-4800 field-emission scanning electron microanalyzer (Hitachi, Tokyo, Japan). The Fourier transform infrared spectra (FTIR) measurements were conducted on a Thermo Scientific Nicolet iS10 (Thermo Fisher, Shanghai, China). The phase structures of samples were analyzed by a Rigaku D/MAX-2500 (Rigaku, Shoshima, Tokyo, Japan). The X-ray photoelectronic spectroscopy (XPS) was collected on a Themno Fisher K-Aepna X-ray photoelectron spectrometer (Themno Fisher, Waltham, MA, USA) with Al Kα (1361eV) source. N_2_ sorption isotherms of samples were analyzed by a Quantachrome instruments autosorb-iQ (Quantachorme, Shanghai, China) at 77 K.

### 2.4. Electrochemical Measurements

The electrochemical experiments were tested by cyclic voltammentry (CV) and galvanostatic charge-discharge in 2 mol L^−1^ H_2_SO_4_ solutions, which were performed on a CHI 760D electrochemical workstation (Themno Fisher, Waltham, MA, USA) in a two-electrode system. The supercapacitors were prepared by employing the PBCs as free-standing electrode, a PP/PE membrane as the separator and a couple of stainless steel sheets as current collectors. In addition, the mass loading of PBCs was 2.5 mg (diameter about 16 mm).

## 3. Results

**Figure 1:**
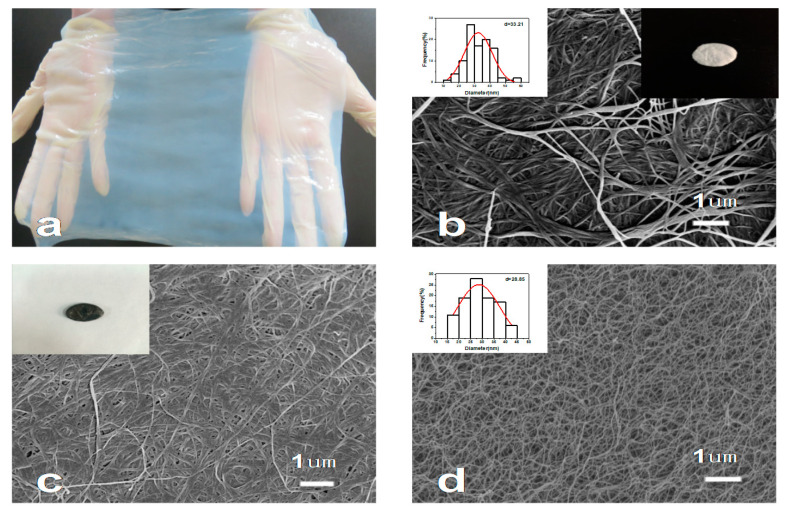
(**a**) Photograph of a bacterial cellulose (BC) pellicle. (**b**) SEM image of the freeze-dried BC pellicle surface (Top right inset: the typical sample, *d* = 20 mm; Top left inset: the fiber diameter distribution). (**c**) SEM image of the pyrolyzed (P)BC surface (inset: the typical sample, *d* = 16 mm). (**d**) SEM image of the inner of PBC (inset: the fiber diameter distribution).

The primitive BC exhibited water-rich morphological characteristics, showing its strong hydrophilicity, which is due to the abundant hydrophilic groups in its network structure (Figure 1a). In the process of freeze-drying, ice directly sublimates into water vapor, which can prevent the collapse of the gel-network at the most extent [27]. The BC is constituted of mutual connective nanofibrils and cross-linked pores, and the average diameter of these nanofibers is about 33.21 nm (Figure 1b). The as-obtained BC aerogels were then pyrolyzed under flowing nitrogen at 800 °C to form PBC, which is a black, superlight conductive aerogel and reserves the inherent highly multihole and ultrafine network nanostructure of BC [28], with an average diameter of 28.85 nm (Figure 1c,d). The decrease in the quality is due to the carbonization of the BC and evaporation of volatiles such as CO, CO_2_, methanol and acetic acid [29].

**Figure 2:**
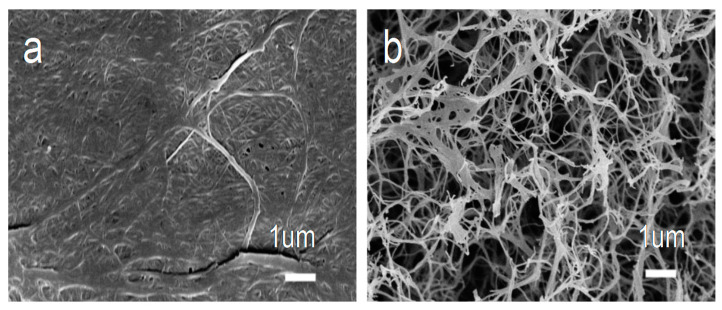
SEM image of (**a**) the surface and (**b**) the inner of N/P/S-PBC-3.

**Figure 3:**
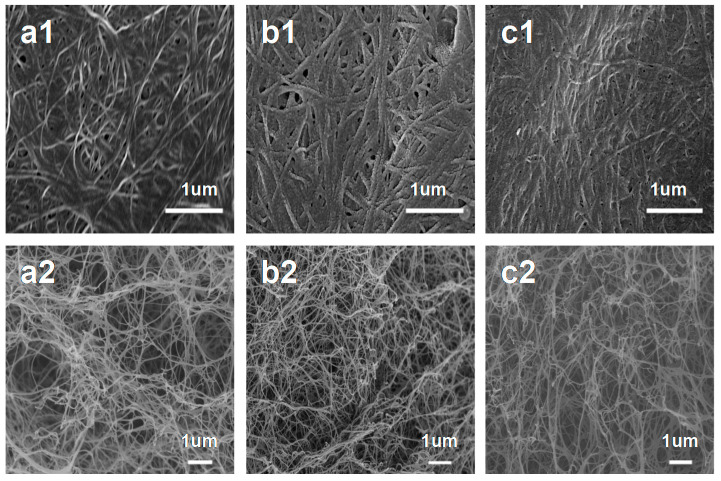
SEM image of (**a1**) the surface and (**a2**) the inner of N/P co-doped pyrolyzed bacterial cellulose (N/P-PBC), (**b1**) the surface and (**b2**) the inner of N/S co-doped pyrolyzed bacterial cellulose (N/S-PBC), (**c1**) the surface and (**c2**) the inner of N/P/S co-doped pyrolyzed bacterial cellulose (N/P/S-PBC).

In the process of impregnation, the high concentration of NH_4_H_2_PO_4_ or (NH_4_)_2_SO_4_ aqueous solution would make the as-prepared bacterial cellulose/NH_4_H_2_PO_4_, bacterial cellulose/(NH_4_)_2_SO_4_ and bacterial cellulose/NH_4_H_2_PO_4_-(NH_4_)_2_SO_4_ slices thin, which made PBCs bond together on the surface and break in inner (Figure 2). In general, the distributions of N/P-PBC, N/S-PBC and N/P/S-PBC nanofibers were uniform and presented an almost perfect network structure (Figure 3). Such nanofibers can increase the interfacial area, and the special network architecture would enable the fast transport of ion and electron in its three-dimensional directions [30].

**Figure 4:**
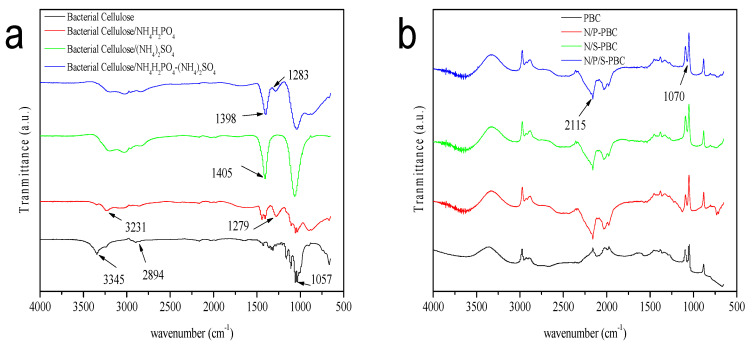
FTIR spectra of (**a**) BC, N/P-BC, N/S-BC, N/P/S-BC and (**b**) PBC, N/P-PBC, N/S-PBC, N/P/S-PBC.

The functional groups in the sample were identified by FTIR spectrum. As Figure 4a shows, the freeze-drying of the BC samples showed several typical vibration bands at 1057 cm^−1^ (skeletal vibrations involving C–O stretching), 2894 cm^−1^ (C–H stretching of CH_2_ groups) and 3345 cm^−1^ (O–H stretching vibration). After impregnating, samples showed several important peaks at 3231 cm^−1^ (stretching of N–H), 1279 cm^−1^ (stretching of P=O) and 1405 cm^−1^ (stretching of –SO_2_–). These results showed that the solvent (NH_4_H_2_PO_4_ or (NH_4_)_2_SO_4_) was combined with the large number of functional groups (–OH or C=O) of BC [31]. After carbonization, the heteroatoms such as H and O were volatilized because of the broken of the C–H, C–O and O–H bonds. Hence, the peaks of more than 3000 cm^−1^ disappeared and the intensity of other peaks was weakened (Figure 4b). In the spectra of N/P-PBC, N/S-PBC and N/P/S-PBC, a peak appeared at 2115 cm^−1^ corresponding to the –NH stretching vibration, which demonstrated the successful introduction of ammonium salt.

**Figure 5:**
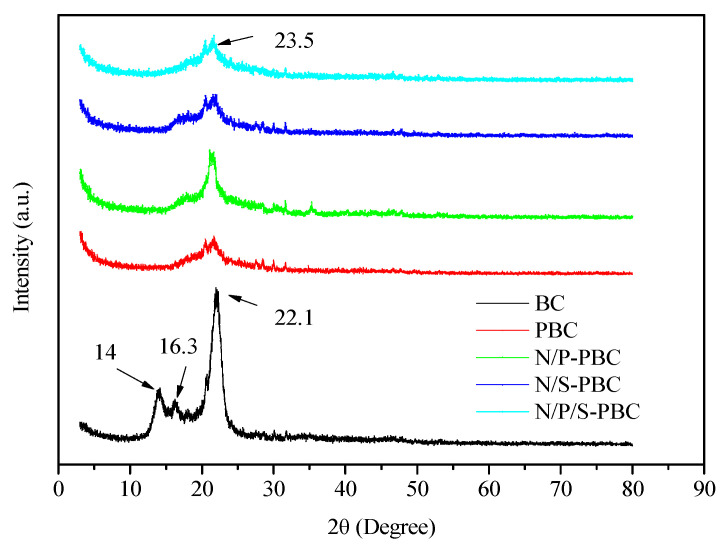
XRD patterns of BC, PBC, N/P-PBC, N/S-PBC and N/P/S-PBC.

The crystallinity and the graphitization degree of samples were characterized by XRD (Figure 5). The three main peaks located at 14°, 16.3° and 22.1° could be assigned to the (110), (110) and (200) diffraction planes of cellulose I structure, respectively [15]. In the XRD patterns of PBCs, a wide and weak diffraction peaks appeared at 23.5°, which may be ascribed to the (002) facets of hexagonal graphitic structure, showing that the sample was amorphous carbon with a lower graphitization degree in the carbonization temperature at 80 °C [32].

**Figure 6:**
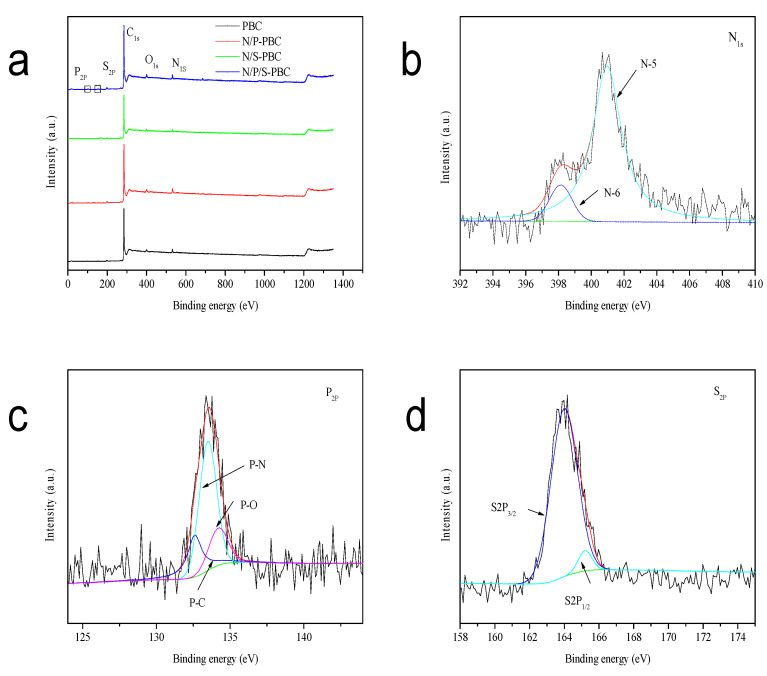
(**a**) XPS survey spectra of the PBC, N/P-PBC, N/S-PBC, N/P/S-PBC and the high-resolution XPS spectra of deconvoluted (**b**) N1s, (**c**) P2p and (**d**) S2p peak of N/P/S-PBC.

It can be seen from X-ray photoelectron spectra (XPS) survey spectra that N, P and S were indeed co-doped into the carbon framework in as-prepared sample (Figure 6a). Here, the survey spectra of N/P/S-PBC is demonstrated and its binding environment is elucidated by the high-resolution XPS spectra (Figure 6b–d). The deconvolution of the high-resolution N1s peak could be separated into two kinds of nitrogen functional groups, pyridinic (N-6, 398.1 eV) and pyrrolic/pyridine (N-5, 400.9 eV), and the high proportion of available N species (N-5) would provide abundant active sites to improve the energy density of supercapacitor (Figure 6b, Table 2) [33]. Furthermore, the high-resolution P2p peak spectrum could be divided into three peaks, 132.6, 133.5 and 134.2 eV, which correspond to P–C binding, P–N binding and P–O binding, respectively (Figure 6c) [34]. In addition, the high-resolution S2p peak spectrum yielded two peaks, 164.0 and 165.2 eV, which correspond to S2p_3/2_ and S2p_1/2_, which illustrated that S atom combined with the C atoms to form C–S–C binding at the edge and defects of the carbon frameworks (Figure 6d) [19]. Besides, the XPS results indicated that 3.9 atom% of nitrogen, 1.22 atom% of phosphorus and 0.6 atom% of sulfur were doped into the N/P/S-PBC (Table 3). It is believed that the N-5, P-C and S-C functionalities can provide more active sites to improve its capacitance, which was confirmed by the remarkable electrochemical performance of those reported materials [35,36].

**Figure 7:**
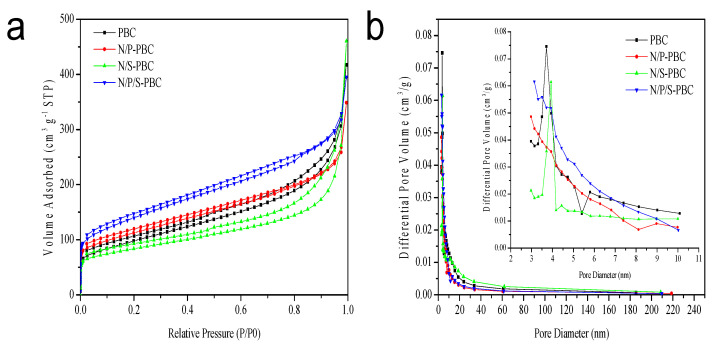
N_2_ adsorption–desorption isotherms (**a**) and pore size distribution (**b**) curves of PBCs.

The porous structures of PBCs were investigated by nitrogen adsorption–desorption isotherms (Figure 7a). In the adsorption curves at a high relative pressure of 0.95, a following sharp increase was observed, resulting from the multilayer adsorption of nitrogen in macropores formed among the carbon nanofibers [32]. The pore size distribution of all the PBCs became narrower with the peaks centered around 3.5 nm (Figure 7b). The BET surface area and pore structure parameters of PBCs calculated from the isotherms are listed in Table 4. The N/P/S-PBC shows a maximum specific surface area of 498 m^2^/g, which attributed to the electric double-layer capacitance. Its pore volume and average pore diameter can reach up to 0.61 cm^3^/g and 3.13 nm. The result reveals that the macropores of network-like structure, mesopores and micropores of N/P/S-PBC will contribute to the diffusion of the electrolyte in the aqueous electrochemical capacitors.

Therefore, after a simple, low-cost solution impregnation method, three elements (N, P and S) were introduced to the bacterial-cellulose-based carbon nanofibers and increased the surface area, which will contribute to the pseudocapacitance and electric double-layer capacitance, respectively. This may be a good way to introduce a functional group to carbon materials.

**Figure 8:**
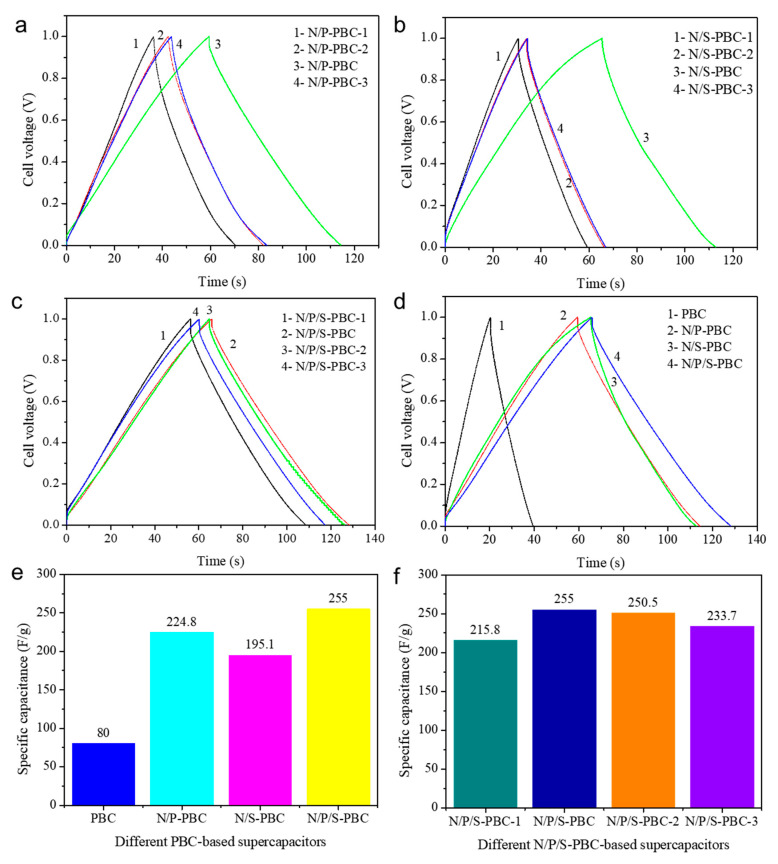
Galvanostatic charge–discharge curves of the different (**a**) N/P-PBC-based supercapacitor, (**b**) N/S-PBC-based supercapacitor, (**c**) N/P/S-PBC-based supercapacitor, (**d**) the different PBCs supercapacitors measured using a two-electrode system in 2.0 M H_2_SO_4_ aqueous electrolyte at the current density of 1.0 A/g and the specific capacitor values of (**e**) different pyrolyzed bacterial cellulose nanofiber-based supercapacitors and (**f**) different N/P/S-PBC-based supercapacitors at the current density of 1.0 A/g.

The electrochemical performances of PBCs were investigated in aqueous electrolyte of 2.0 M H_2_SO_4_ with a two-electrode system. The supercapacitors constructed include pure PBC supercapacitors, N/P-PBC-based supercapacitors, N/S-PBC-based supercapacitors and N/P/S-PBC-based supercapacitors. Their galvanostatic charge–discharge curves at 1.0 A/g show that the specific supercapacitance *C*_s_ (Equation (S1), Supporting Information) of N/P/S-PBC-based supercapacitors is higher than the other supercapacitors (Figure 8a–c). Meanwhile, compared with other N/P/S-PBC-based supercapacitors, the N/P/S-PBC supercapacitors shows a higher value of *C*_s_ (Figure 8d,e), and find that increasing the concentration of the (NH_4_)_2_SO_4_/NH_4_H_2_PO_4_ aqueous solution from 0.05/0.025 to 0.1/0.025 and 0.05/0.05, then to 0.1/0.05, will lead to a change in *C*_s_ from 215.8 to 255.0 and 250.5, then to 233.7 F/g at 1.0 A/g, respectively (Figure 8f). These enhanced electrochemical properties could be ascribed to the appropriate amounts of nitrogen, phosphorus and sulfur incorporated into carbon network and the increased BET surface area. However, the decrease in *C*_s_ may be due to the corrosion of the high concentration aqueous solution which damage the network structure of nanofiber (Figure 2b). Hence, the performance of the N/P/S-PBC supercapacitor was further studied.

**Figure 9:**
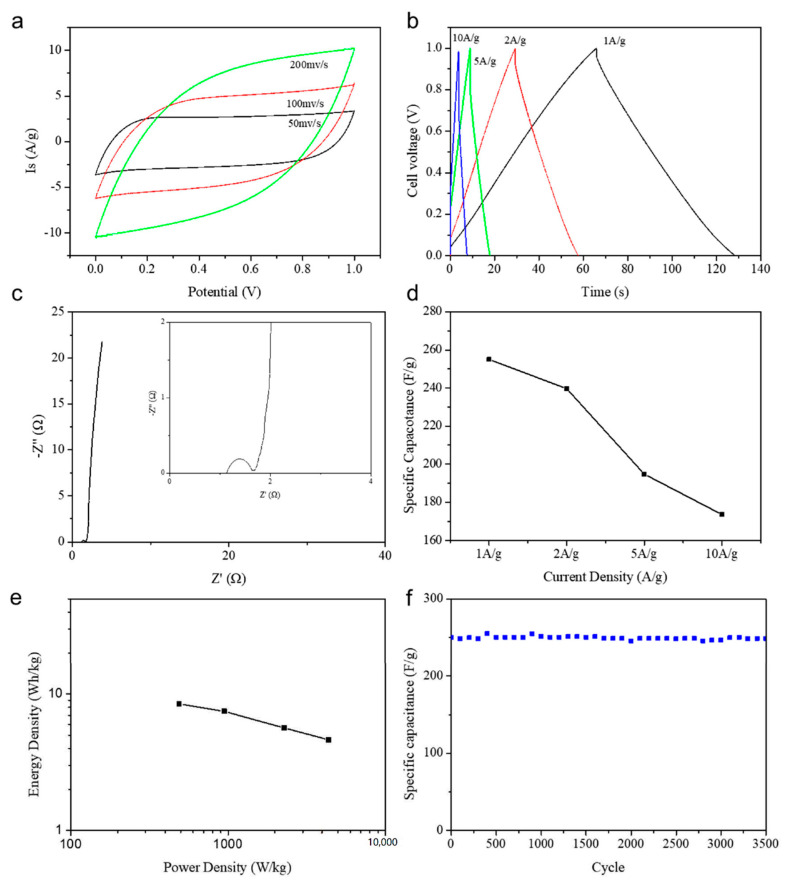
Electrochemical properties of the N/P/S-PBC//N/P/S-PBC supercapacitor measured using a two-electrode system in a 2.0 M H_2_SO_4_ aqueous electrolyte. (**a**) CV curves at different scan rates. (**b**) Galvanostatic charge–discharge curves at different current densities. (**c**) Electrochemical impedance spectra. (**d**) Variation in specific capacitance against current density. (**e**) Ragone plot. (**f**) Cycling performance at a current density of 1.0 A/g.

In Figure 9a, with the scan rate increasing from 50 to 200 mV/s at a potential window of 1.0 V, the CV curves of N/P/S-PBC supercapacitors maintain quasi-rectangular shape, indicating its high-rate capability and good capacitive behavior. The charge–discharge curve of the N/P/S-PBC supercapacitor exhibits a nearly symmetrical triangle and slightly nonlinear sloping potential profiles in a larger current density, which indicates that the supercapacitor has the properties of fast charge–discharge response and low internal resistance, and the redox reactions occur on the surface of the N/P/S-PBC electrode (Figure 9b) [37]. Electrochemical impedance spectroscopy (EIS) can give vast quantities of information regarding the internal resistance of the electrode material and resistance between the electrode and electrolyte. A Nyquist impedance spectrum of the N/P/S-PBC supercapacitor is presented in Figure 9c; it can be clearly seen that a semicircle is observed at the high-frequency region and an erect slant line at the low-frequency region. It is apparent that the charge-transfer resistance (Rct) for N/P/S-PBC is about 1.54 Ω, which is crucial for enhancing the rate capability of the supercapacitors [38]. Furthermore, it also can see from Figure 9d that about 74% of the initial specific capacitance retention ratio is obtained at 10 A/g, which is consistent with the results of the EIS.

**Figure 10:**
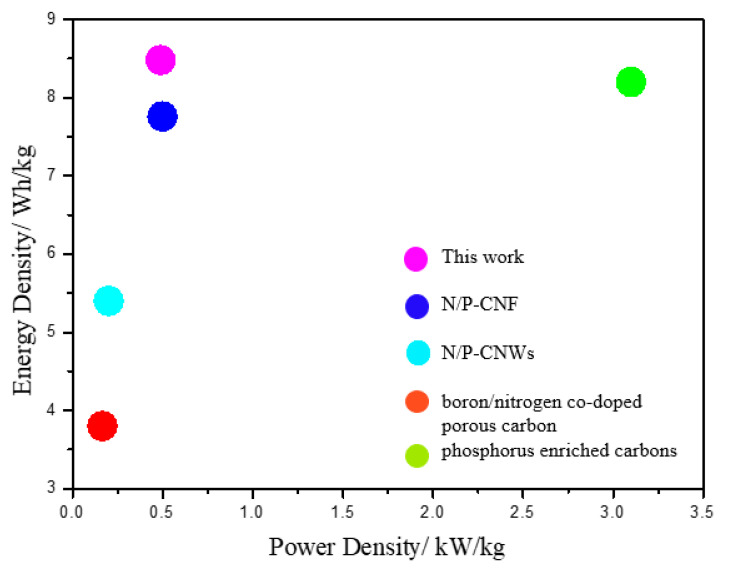
Comparision of the N/P/S-PBC surpercapacitor with other heteroatom-doped carbon-based surpercapacitor [20,28,37,39].

The power density (*P*) and energy density (*E*) are calculated to estimate the performance of the N/P/S-PBC supercapacitor (Equations (S2,S3), Supporting Information). In Figure 9e, the N/P/S-PBC supercapacitor shows a high energy density about 8.48 Wh/kg with a power density of 489.45 W/kg, which is higher than other heteroatom-doped, carbon-based supercapacitors (Figure 10). The high energy density is mainly attributed to the suitable co-doped of nitrogen/phosphorus/sulfur and increased surface area in the N/P/S-PBC electrode, which contribute to the pseudocapacitance and electric double-layer capacitance, respectively. The cycling stability is also a vital characteristic and was performed by a repeating galvanostatic charge–discharge test at 1.0 A/g. The result in Figure 9f shows that its specific capacitance value exhibits a slight change after 3500 cycles, which confirms that the as-prepared surpercapacitor has the merit of practical applications.

## 4. Conclusions

N/P/S-PBC was successfully manufactured via employing a low-cost, eco-friendly bacterial cellulose as carbon nanofiber. When 3.9 atom% of nitrogen, 1.22 atom% of phosphorus and 0.6 atom% of sulfur co-doped into PBC, the specific capacitance for N/P/S-PBC at 1.0 A/g is about 255 F/g and the N/P/S-PBC supercapacitor’s energy density is about 8.48 Wh/kg with a power density of 489.45 W/kg. The excellent electrochemical properties of N/P/S-PBC are due to the perfect three-dimensional structure of the PBC aerogels and the synergistic interaction of heteroatoms when co-doped, which contribute to the electric double-layer capacitance and pseudocapacitance, respectively. Therefore, the simple and convenient method could be scaled up for industrial applications in large-scale supercapacitor electronics.

## Figures and Tables

**Table 1:** The designation of samples prepared in this work.

Sample Codes	Concentration of NH_4_H_2_PO_4_ Solution (mol/L)	Concentration of (NH_4_)_2_SO_4_ Solution (mol/L)
N/P-PBC-1	0.02	0
N/P-PBC-2	0.05	0
N/P-PBC	0.1	0
N/P-PBC-3	0.2	0
N/S-PBC-1	0	0.005
N/S-PBC-2	0	0.01
N/S-PBC	0	0.05
N/S-PBC-3	0	0.1
N/P/S-PBC-1	0.05	0.025
N/P/S-PBC	0.1	0.025
N/P/S-PBC-2	0.05	0.05
N/P/S-PBC-3	0.1	0.05

**Table 2:** Area percentage of N1s, P2p and S2p peaks in high resolution XPS spectra for N/P/S-PBC.

Sample	Area Percentage (%)						
	N-5	N-6	P-C	P-N	P-O	S_2p3/2_	S_2p1/2_
N/P/S-PBC	89.18	10.82	23.37	58.20	18.43	94.17	5.827

**Table 3:** The contents of nitrogen, phosphorous, sulfur and oxygen element (atom ratio, derived from XPS analysis) of different PBCs (the deviation of all test data is within 5%).

Sample	N1s	P2p	S2p	O1s
PBC	1.4	0	0	1.85
N/P-PBC	3.67	2.41	0	3.57
N/S-PBC	3.27	0	1.34	2.6
N/P/S-PBC-1	3.64	0.96	0.71	2.82
N/P/S-PBC	3.90	1.22	0.62	3.97
N/P/S-PBC-2	3.83	0.82	1.14	3.61
N/P/S-PBC-3	4.18	1.34	1.20	3.84

**Table 4:** Pore parameters of the sample PBCs. (Calculated total surface *S_BET_*, total pore volume *V_T_*, average pore diameter *d_M_*).

Sample	*S_BET_* (m^2^/g)	*V_T_* (cm^3^/g)	*d_M_* (nm)
PBC	350	0.65	3.71
N/P-PBC	397	0.54	2.97
N/S-PBC	296	0.71	3.93
N/P/S-PBC	498	0.61	3.13

## Supplementary Materials

The following are available online at https://www.mdpi.com/2079-4991/10/10/1912/s1. S1. Equations.
