# Supplementary material for: Nitrogen, Phosphorus and Sulfur Co-Doped Pyrolyzed Bacterial Cellulose Nanofibers for Supercapacitors
Source: Nanomaterials (Basel). 2020 Sep 25;10(10):1912. doi: 10.3390/nano10101912 (PMC7599491; doi:10.3390/nano10101912)
Supplement: Supplementary file 1 [file nanomaterials-10-01912-s001.pdf]

Supplementary Materials: Nitrogen, Phosphorus and Sulfur Co-Doped Pyrolyzed Bacterial Cellulose Nanofibers for Supercapacitors

Zheng Li, Yaogang Wang, Wen Xia, Jixian Gong, Shiru Jia and Jianfei Zhang

S1. Equations

Specific capacitance values of the electrode composites were calculated from the galvanostatic discharge process according to the following Equation [1]:

$$C_s = \frac{4I \times \Delta t}{m \times \Delta V}, \tag{S1}$$

where *C*s (F g^{-1}) represents specific capacitance of the electrode material, *I* (A) corresponds to the discharge current, Δ*V* (V) is the potential change within the discharge time Δ*t* (s), and *m* (g) refers to the total mass of active material on the two electrodes of the capacitor.

The power density (*P*) and energy density (*E*), were calculated using Equations (S2,S3) [2].

$$E = \frac{1}{8} \times C_s \times (\Delta V)^2, \tag{S2}$$

$$P_{av} = \frac{E}{\Delta t}. \tag{S3}$$

where *C*s (F g^{-1}) represents the specific capacitance of the electrode material measured from the Equations (S1), Δ*V* (V) refers to the potential change within the discharge time Δ*t* (s), *E* (J g^{-1}) is the energy density, *P*av (W g^{-1}) is the average power density.

References

1. Chen, L.; Huang, Z.; Liang, H.; Gao, H.; Yu, S. Three-Dimensional Heteroatom-Doped Carbon Nanofiber Networks Derived from Bacterial Cellulose for Supercapacitors. *Adv. Funct. Mater* **2014**, 24, 5104-5111.
2. Chen, L.; Huang, Z.; Liang, H.; Yao, W.; Yu, Z.; Yu, S. Flexible all-solid-state high-power supercapacitor fabricated with nitrogen-doped carbon nanofiber electrode material derived from bacterial cellulose. *Energy Environ. Sci.* **2013**, 6, 3331-3338.
